# Changes in functional connectivity and GABA levels with long-term motor learning

**DOI:** 10.1016/j.neuroimage.2014.11.032

**Published:** 2015-02-01

**Authors:** Cassandra Sampaio-Baptista, Nicola Filippini, Charlotte J. Stagg, Jamie Near, Jan Scholz, Heidi Johansen-Berg

**Affiliations:** a Oxford Centre for Functional MRI of the Brain (FMRIB), Nuffield Department of Clinical Neurosciences, University of Oxford, John Radcliffe Hospital, Headington, Oxford OX3 9DU, UK; b Department of Psychiatry, University of Oxford, Warneford Hospital, OX3 7JX, UK; c Mouse Imaging Centre, Hospital for Sick Children, 25 Orde Street, Toronto, Ontario M5T 3H7, Canada

**Keywords:** Plasticity, Functional connectivity, GABA, Motor learning

## Abstract

Learning novel motor skills alters local inhibitory circuits within primary motor cortex (M1) (Floyer-Lea et al., 2006) and changes long-range functional connectivity (Albert et al., 2009). Whether such effects occur with long-term training is less well established. In addition, the relationship between learning-related changes in functional connectivity and local inhibition, and their modulation by practice, has not previously been tested.

Here, we used resting-state functional magnetic resonance imaging (rs-fMRI) to assess functional connectivity and MR spectroscopy to quantify GABA in primary motor cortex (M1) before and after a 6 week regime of juggling practice. Participants practiced for either 30 min (high intensity group) or 15 min (low intensity group) per day. We hypothesized that different training regimes would be reflected in distinct changes in brain connectivity and local inhibition, and that correlations would be found between learning-induced changes in GABA and functional connectivity.

Performance improved significantly with practice in both groups and we found no evidence for differences in performance outcomes between the low intensity and high intensity groups. Despite the absence of behavioral differences, we found distinct patterns of brain change in the two groups: the low intensity group showed increases in functional connectivity in the motor network and decreases in GABA, whereas the high intensity group showed decreases in functional connectivity and no significant change in GABA. Changes in functional connectivity correlated with performance outcome. Learning-related changes in functional connectivity correlated with changes in GABA.

The results suggest that different training regimes are associated with distinct patterns of brain change, even when performance outcomes are comparable between practice schedules. Our results further indicate that learning-related changes in resting-state network strength in part reflect GABAergic plastic processes.

## Highlights

• Long-term learning modulated functional connectivity.
• Changes in functional connectivity correlated with performance outcome.
• Long-term learning decreased GABA levels.
• Learning-related changes in functional connectivity correlated with changes in GABA.

## Introduction

Learning of demanding novel motor skills, acquired over several training sessions, induces structural and functional plasticity in the brain (e.g., Dayan and Cohen, 2011; Doyon et al., 2009; Sampaio-Baptista et al., 2014; Scholz et al., 2009). The amount of practice undertaken modulates structural changes associated with long-term motor learning (Sampaio-Baptista et al., 2014) and influences the functional networks recruited for task performance (Doyon and Ungerleider, 2002). Recently, resting-state fMRI has been used to show that motor learning changes the resting functional connectivity of the brain. For instance, 11 min of motor training increased the strength of the fronto-parietal and the cerebellum resting state networks (RSNs) (Albert et al., 2009). Another study hinted that the strength of the motor RSN varies with different learning stages (Ma et al., 2011). However, the effect of different practice schedules on functional brain connectivity has not previously been tested. Also, it is not clear what these changes reflect in terms of neurophysiological mechanisms.

Previous studies have suggested that RSNs may be driven in part by activity within GABAergic networks (Kapogiannis et al., 2013; Stagg et al., 2014). One study demonstrated a negative correlation between GABA levels in the posteromedial cortex and the strength of the default mode network (Kapogiannis et al., 2013) while another has shown a negative relationship between local GABA concentrations within the primary motor cortex (M1) and motor RSN strength (Stagg et al., 2014). These findings are in line with converging evidence from simulation and MEG studies suggesting that connectivity within the motor RSN can be related to fluctuations in beta and gamma frequency oscillations within M1, which are known to be modulated by local GABAergic activity (Brookes et al., 2011; Cabral et al., 2011; Hall et al., 2011).

The role of GABA in modulating learning-related synaptic changes is well described in animal models (Donato et al., 2013; Froc et al., 2000; Trepel and Racine, 2000). Similarly, previous spectroscopy studies in humans have shown a decrease in GABA levels in response to short-term changes in sensorimotor experience (Floyer-Lea et al., 2006; Levy et al., 2002). However, there are no reports on modulation of GABA with long-term learning in humans.

Here, we test the relationship between learning-related changes in network-level functional connectivity and local inhibition, and their modulation by different practice schedules. We have manipulated the amount of juggling training per day to test how the strength of the motor network and GABA levels is modulated by different amounts of juggling practice.

We hypothesize that where circuits have been strengthened by decreases in local inhibitory tone we will see increases in strength of corresponding RSNs and decreases in local GABA. Where there has been a net decrease in circuit strength, for instance due to increased efficiency, we would predict a decrease in RSN strength. We further hypothesize that GABA concentration change in response to motor learning will be negatively correlated with change in the strength of the motor resting-state network (Kapogiannis et al., 2013; Stagg et al., 2014).

## Methods

### Participants and experimental design

Sixty four participants (mean age 23.8, standard deviation 3.5; 31 female) gave their informed consent to participate in the study in accordance with local ethics committee approval (Oxfordshire REC B 07/Q1605/65).

Of these, forty four naïve participants were randomly assigned to one of two groups: a high intensity training group that practiced juggling for 30 min per day or a lower intensity group that practiced for 15 min per day. Four participants in the low intensity group dropped out of the study (high intensity n = 22; low intensity n = 18). Both groups trained for 5 days a week for 6 weeks and were scanned at baseline, after 6 weeks of training (Post 1) and again after a subsequent 4 week period (Post 2), during which participants did not juggle. Results from other imaging modalities acquired in the same participants have been reported previously (Sampaio-Baptista et al., 2014).

In addition, a control group of 20 participants was scanned twice 6 weeks apart but received no juggling training.

FMRI data was acquired in 20 participants in the high intensity group, 16 in the low intensity group and 20 in the control group. MRS data was acquired in 20 high intensity group participants (note that 2 of these participants did not have fMRI data acquired), in 16 low intensity training participants and 19 control participants because the Specific Absorption Rate (SAR) limit was exceeded.

### Behavioral assessment

Behavioral assessment is described elsewhere (Sampaio-Baptista et al., 2014). Briefly, participants in the training groups had a group lesson on the first training day, where the simplest juggling pattern – the ‘3 ball cascade’ – was taught. Subsequently, subjects practiced daily at home for 29 days. Participants filmed each home training session using a webcam and were required to upload their training videos to a secure website daily. Volunteers who mastered the 3-ball cascade before the end of the training period were encouraged to practice more advanced juggling pattern like the 3-ball reverse cascade. After the training period, participants stopped juggling for 4 weeks.

Final daily scores were derived from the experimenter rating of each of the 29 training videos per participant in a 0–10 scale (0: 2 balls; 1: 1 cycle of 3-ball cascade; 2: 2 cycles; 3: 3 cycles; 4: 5–10 s of sustained 3-ball cascade; 5: 10–20 s; 6: 20–30 s; 7: > 30 s; 8: > 60 s; 9: > 60 s and at least one other pattern for < 60 s; 10: > 60 s and at least one other pattern for > 60 s) (Sampaio-Baptista et al., 2014; Scholz et al., 2009). A logarithmic curve was fitted to each participant's daily scores and the slope of the curve (learning rate) was calculated.

### MRI acquisition

Scanning was performed at the University of Oxford using a 3 Tesla Siemens Trio scanner with a 12-channel head-coil. Whole-brain fMRI was performed using a gradient echo EPI sequence while participants were at rest with eyes open (TR = 2000 ms, TE = 28 ms, flip angle = 89°, field of view = 224 mm, voxel dimension = 3 × 3 × 3.5 mm, acquisition time = 6 min 4 s). FMRI data was acquired in 20 participants in the high intensity group, 16 in the low intensity group and 20 in the control group.

We acquired one axial T1-weighted anatomical image per session using a MPRAGE sequence (TR = 20.4 ms; TE = 4.7 ms; flip angle = 8°; voxel size = 1 × 1 × 1 mm^3^).

Metabolite concentrations in the motor hand representation were assessed using Spin-Echo full intensity acquired localized (SPECIAL; TR = 3000 ms; TE = 8.5 ms; flip angle = 90°; voxel size = 20 × 20 × 20 mm^3^; total scan time = 9 min 48 s) (Mekle et al., 2009). Data were acquired from a 20 × 20 × 20 mm voxel placed manually over the left precentral knob (Yousry et al., 1997). MRS data was acquired in 20 high intensity group participants (note that 2 of these participants did not have fMRI data acquired), in 16 low intensity training participants and 19 control participants because the Specific Absorption Rate (SAR) limit was exceeded.

### MRI analysis

MRI data analysis was carried out using FSL tools (www.fmrib.ox.ac.uk/fsl). Resting-state fMRI was analyzed with Multivariate Exploratory Linear Optimized Decomposition into Independent Components (MELODIC) (Beckmann et al., 2005). MELODIC is a data driven method that identifies components containing brain areas with time-courses correlated with each other that are independent of other components. Standard preprocessing included correction for head motion, brain extraction, spatial smoothing using a Gaussian kernel of full-width at half-maximum (FWHM) of 6 mm, and high-pass temporal filtering equivalent to 150 s (0.007 Hz). FMRI volumes were registered to the individual's structural scan using boundary-based registration (BBR) (Greve and Fischl, 2009) and then to standard space with FMRIB's Nonlinear Image Registration Tool (FNIRT) (Andersson et al., 2007).

Preprocessed functional data containing 180 time points per subject were temporally concatenated across subjects to create a single 4D dataset. This 4D dataset, containing all participants and all scans, was then used as an input to MELODIC. MELODIC was used to identify previously described ‘canonical’ resting-state networks at a group level (Beckmann and Smith, 2004).

Next, a dual-regression approach was applied to each group level RSN of interest (Filippini et al., 2009), in order to calculate individual subject measures of the strength of each RSN. The steps involved in dual regression were as follows:

1. The spatial map associated with each group-ICA of interest was regressed back to individual subject data in order to find the time-courses for each subject associated with each group-level component;
2. These individual subject time-courses were then used as regressors to identify subject specific spatial maps in which voxel values represent the strength of association with the particular group ICA of interest;
3. The group mean ICA spatial map for each component of interest was used as a region of interest and applied to the individual subject maps created in step 2, in order to calculate the mean value for that network for each participant. This value corresponds to the network ‘strength’ i.e. the higher the value the more correlated are the areas within the network (Stagg et al., 2014).

GABA concentration was calculated automatically with LCModel (Provencher, 2001) using a basis set consisting of 41 simulated metabolite model spectra. All metabolite concentrations are given as a ratio to total creatine (creatine + phosphocreatine). Metabolite concentrations with a Cramer Rao Lower Bound (CRLB) > 15%, a measure of reliability of the LCModel fit, were excluded from analysis (2 in control group (total n = 17), 2 in low intensity group (total n = 14), 2 in high intensity group (total n = 18)).

FMRIB's Automated Segmentation Tool (FAST) was used to calculate relative quantities of gray matter (GM) and white matter (WM) within the voxel based on the high-resolution T1-weighted anatomical images. The GABA concentrations were corrected for the proportion of GM volume within the voxel [divided by [GM] / ([GM] + [WM] + [CSF])] and creatine was corrected for the proportion of total brain tissue volume within the voxel [divided by ([GM] + [WM]) / ([GM] + [WM] + [CSF])] (Stagg et al., 2009).

### Statistical analysis

SPSS software (Version 21.00) was used to analyze juggling performance, resting-state network strength and neurotransmitter concentration data.

Normality was tested with Shapiro–Wilk for all data before statistical testing. We tested for juggling performance differences over time (30 days) between groups with Mixed-Design ANOVA (MD-ANOVA). When Mauchly's test of sphericity was statistically significant, Greenhouse–Geisser F-test was used and the respective degrees of freedom are reported. Additionally, a *t*-test was used to test for differences in learning rate between groups. Note that although behavioral results from this experiment have been reported previously (Sampaio-Baptista et al., 2014), here we are reporting the behavioral results for the specific participants that had MRS or resting fMRI acquired.

**Fig. 1:**
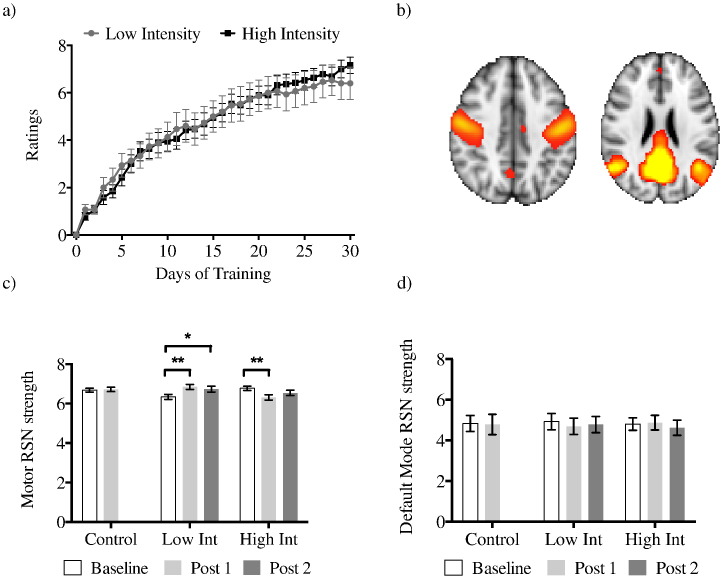
a) Average performance ratings for each group per day. There is a significant effect of day (F_(3.933,129.795)_ = 142.2, p = 0.00001) but no significant interaction effect (F_(3.933,129.795)_ = 0.999, p > 0.1) or significant differences between groups (F_(1,33)_ = 0.005, p > 0.1). b) Motor resting-state network (left). Default mode network (right). c) Motor resting-state network strength. The low intensity group shows significant increases between Baseline and Post 1 (**t_(15)_ = 3.283, p = 0.005) and a trend for increases between Baseline and Post 2 (*t_(15)_ = 2.347, p = 0.033). There is significant decrease in motor RSN strength between Baseline and Post 1 for the high intensity group (**t_(19)_ = 2.787, p = 0.012). There was no difference in motor RSN strength between Baseline and Post 1 for the control group (t_(19)_ = 0.243, p > 0.1). d) Default mode network strength. Training had no significant effect on the default mode RSN. Bars represent standard error. *uncorrected, **survives Bonferroni correction, p < 0.016.

We tested for RSN strength differences between group (high, low, control), time-point (Baseline and Post 1) and RSN (motor, default mode) and interaction effects using MD-ANOVA. Note that only baseline 1 and Post 1 timepoints could be included in the initial ANOVA as only these timepoints were collected for controls. The motor RSN was considered the network of interest and the default mode RSN was used as a control as it is largely spatially distinct from the motor RSN (Figs. 1a, b). If significant interactions were found then these were followed up with post-hoc repeated-measures (RM) ANOVAs or t-tests as appropriate. Differences in Post 1 RSN strength between groups were tested using ANCOVA to account for baseline differences, with baseline measures as the covariate covariate and Post 1 RSN strength as the dependent variable.

For post-hoc t-tests within the training groups, all 3 timepoints (baseline, Post 1, Post 2) were considered. Bonferroni corrections were performed when appropriate.

We also tested for partial correlations between RSN strength change and performance change, while using baseline RSN as a covariate. Normality was tested with Shapiro–Wilk and, when the data were significantly non-parametric, Spearman tests were used, otherwise Pearson's R was used (p < 0.05, 2-tail). We tested for differences in correlation strength using Fisher's r to z (2-tail).

A MD-ANOVA was used to test for differences between group (low, high, control) and time-point (Baseline and Post 1) and interaction effects for GABA concentration. Differences in Post 1 GABA between groups were tested using ANCOVA to account for baseline differences, with baseline measures as the covariate and Post 1 GABA as the dependent variable. For post-hoc t-tests within the training groups, all 3 timepoints (baseline, Post 1, Post 2) were considered. Bonferroni corrections were performed when appropriate.

Finally, we tested if GABA change was negatively correlated with motor RSN strength change (p < 0.05, 1-tail, as the direction of this relationship was predicted a priori (Kapogiannis et al., 2013; Stagg et al., 2014)).

## Results

All participants were able to do 3 continuous 3-ball cascade cycles after 6 week training. 5 participants in the high intensity group and 4 in the low intensity group fully mastered the 3-ball cascade and went on to learn more advanced patterns such as the reverse 3-ball cascade. We first investigated differences in performance scores between the low intensity group and the high intensity group throughout the 30 days of juggling. Average juggling performance improved for both groups over time (main effect of day (F_(3.933,129.795)_ = 142.2, p = 0.00001)), but there was no difference between groups (main effect of group (F_(1,33)_ = 0.005, p > 0.1)) or interaction between day and group (F_(3.933,129.795)_ = 0.999, p > 0.1) (Fig. 1a). The two training groups did not differ in rate of learning (slope) (t_(34)_ = 0.758, p > 0.1). In summary, daily practice improved juggling performance but the amount of practice per day did not have any significant effect on performance outcomes.

We went on to test whether the observed improvement in behavior with practice could be associated with changes in resting brain activity (Fig. 1b). Long-term learning altered resting brain activity between the Baseline and Post 1 scans in a network specific manner. A MD-ANOVA revealed a significant main effect of network (F_(1,53)_ = 921.158, p = 0.00001), a trend for an interaction effect between time and group (F_(2,53)_ = 3.101, p = 0.053), and a group × time × network interaction (F_(2,53)_ = 9.182, p = 0.00037). To further investigate these results we ran post-hoc MD-ANOVAs for each RSN separately.

Training had no significant effect on the default mode RSN, whereas for the motor RSN, we found a significant interaction between time and group (F_(2,53)_ = 9.176, p = 0.000379) but no significant main effect of time (F_(1,53)_ = 0.105, p > 0.1) or group (F_(2,53)_ = 0.880, p > 0.1) (Figs. 1c, d).

We then used an ANCOVA to account for any differences in baseline by using baseline measures as a covariate. When comparing Post 1 RSN strength between groups we found a significant effect of group (F_(2,53)_ = 5.154, p = 0.009). Post-hoc tests confirmed that this effect was driven by increases in RSN strength in the low intensity group and decreases in the high intensity group (Fig. 1c). There was no difference in motor RSN strength between Baseline and Post 1 for the control group (t_(19)_ = 0.243, p > 0.1) (Fig. 1c).

**Fig. 2:**
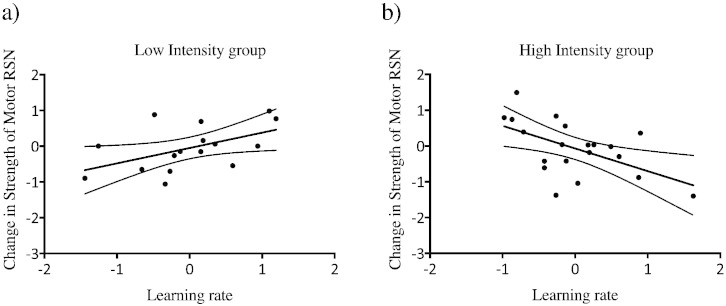
a) There is a positive correlation between the low intensity group increase in motor RSN strength and learning rate (*p = 0.04). b) Significant negative correlation between learning rate and motor RSN strength decrease in the high intensity group (**p = 0.01). The residuals of the partial correlation are plotted. The two correlations are significantly different from each other (z = − 3.21, p = 0.0013). *uncorrected, **survives Bonferroni correction, p < 0.025.

Next we tested whether the training-related change in motor RSN strength could be related to performance level by using a partial correlation with baseline RSN strength as a covariate. We found a significant *negative* correlation between the decrease in motor RSN strength in the high intensity group and learning rate (Pearson r = − 0.549; p = 0.01) (Fig. 2b) and a *positive* correlation between increase in motor RSN strength and learning rate in the low intensity group (Pearson r = 0.513, p = 0.04) (Fig. 2a). To compare these two correlations directly we used Fisher's test and found that the difference between the two correlations was significant (z = − 3.21, p = 0.0013), suggesting that the relationship between RSN strength change and performance differed between practice groups.

**Fig. 3:**
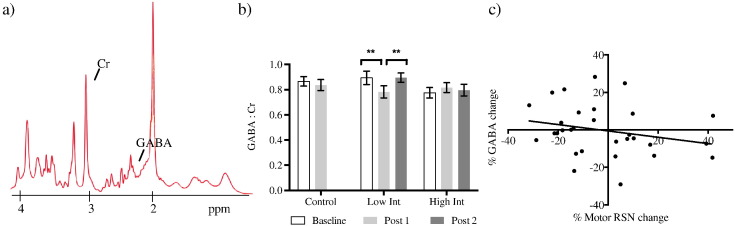
a) Representative MRS spectrum. b) GABA: creatine throughout time in each group. There is a significant difference between Baseline and Post 1 (**t_(13)_ = 3.899, p = 0.002) and Post 1 and Post 2 (**t_(13)_ = 3.075, p = 0.009) for the low intensity group only. **survives Bonferroni correction p < 0.016. c) GABA concentration change is negatively correlated with motor RSN change after learning (Spearman r = − 0.326, p = 0.039). Error bars represent SEM.

We then investigated the effects of training on GABA levels within the primary motor cortex (Fig. 3a). There was an interaction effect between time and group (F_(2,46)_ = 4.655, p = 0.014) but no main effect of time (F_(1,46)_ = 2.583, p > 0.1) nor group (F_(1,46)_ = 0.597, p > 0.1). We used an ANCOVA to account for any differences in baseline. When comparing the Post 1 GABA between groups we found a significant effect of group (F_(2,46)_ = 3.942, p = 0.043). Post-hoc t-tests showed that these effects were driven by a decrease in GABA with learning within the low intensity group (Fig. 3b). There were no differences between Baseline and Post 1 for the control group (t_(16)_ = 0.695, p > 0.1) (Fig. 3b).

Across all jugglers, we found that the change in GABA was negatively correlated with motor RSN strength change (Spearman r = − 0.326, p = 0.039; 1-tail), when GABA decreased the motor RSN strength increased (Fig. 3c). We did not find any significant correlations between GABA change and motor RSN change separately for each group.

## Discussion

This study examined the changes induced by long-term learning of a complex motor task on local inhibitory tone and resting brain connectivity. Although behavioral performance was comparable between two groups that practiced for different amounts of time per day, we found significant differences in brain change between groups: subjects who performed a low intensity practice schedule showed *increases* in motor network connectivity and decreases in GABA whereas those who underwent a high intensity training regimen showed *decreases* in connectivity within the motor RSN and no significant change in GABA. Further, there were significant relationships between performance outcomes and motor RSN strength change, but the direction of these correlations varied between groups: in the high intensity group better performance was associated with greater *decreases* whereas in the low intensity group better performance tended to be associated with greater *increases* in functional connectivity.

The increased motor RSN strength observed in the low intensity group echoes previous reports of increases seen after a single session of short term motor learning (Albert et al., 2009). The differences we observed between the low and high intensity groups are consistent with a previous study of long-term finger sequence learning, which found an increase in functional connectivity between M1 and S1 structures in the first 2 weeks of learning followed by a decrease in connectivity between these regions between week 2 and week 4 (Ma et al., 2011). In the current study we also included a follow-up scan, 4 weeks after the end of training, and found that the increased motor RSN strength detected in the low intensity group was still present at this timepoint, suggesting that it reflects a persistent change in functional connectivity that does not require ongoing practice to be maintained.

One interpretation of the patterns observed here and in previous studies is that lower amounts of practice rely on increasing the strength of previously established functional connections, whereas higher amounts of practice result in increased efficiency (Penhune and Steele, 2011; Toni et al., 1998). These results show interesting parallels to our recent structural findings in an overlapping sample of participants: different amounts of juggling practice resulted in *decreased* GM volume in premotor areas and DLPFC in the low intensity group (that correlated with performance) and in *increased* GM volume in the high intensity group (which also correlated with performance) (Sampaio-Baptista et al., 2014). Both structural and functional measures show relationships with practice and performance in this training task, in overlapping regions (premotor cortex), but also in distinct brain regions (DLPFC), suggesting that there are common underlying drivers for structural and functional change in the motor areas (Sampaio-Baptista et al., 2014). In the previous study, based on the structural results, we hypothesized that different amounts of practice would elicit different cellular mechanisms (Sampaio-Baptista et al., 2014). The current study offers further evidence that lower amounts of practice might elicit pruning (as evidenced by a decrease in GM volume) and rely mostly on previously established functional connections by increasing their strength resulting in increased functional connectivity, while higher amounts of practice might cause formation of new connections (and increases in GM volume) and lead to increased circuit efficiency, reflected here by decreased functional connectivity.

A previous study using a balancing task reported a correlation between performance and changes in resting fMRI signal in the left medial parietal cortex after participants practiced the task once a week throughout 6 weeks (Taubert et al., 2011). These two studies differ in terms of training task and intensity as well as the resting fMRI measures used. For example, the whole-body balancing task used by Taubert et al. is likely to engage proprioceptive processing and that may be why effects there were found in the medial parietal cortex. Although the different measures of functional connectivity used in that study make it hard to directly relate to our results, both studies lend support to the idea that long-term training alters resting brain connectivity in a way that relates to changes in structure (Sampaio-Baptista et al., 2014; Taubert et al., 2011).

An alternative way of thinking about the dissociations found in the current study is that, although both groups have practiced over six weeks, the different daily intensities of practice mean that individuals in the two groups are effectively at different stages of learning (Dayan and Cohen, 2011; Penhune and Steele, 2011). However, to directly test this possibility would require further studies in which more timepoints were used to interrogate learning and brain change at different stages during the 6 week training protocol. One prediction is that the 30 minute group at 3 weeks would appear similar (in terms of GABA and resting fMRI measures) to the 15 minute group at 6 weeks. Future studies using more scanning timepoints could explore the evolution of these changes over time to assess how quickly they arise with training.

The lack of significant performance differences between groups with different practice schedules is notable. However, in our previous studies, using a fixed training protocol identical to the high intensity protocol employed here, we also found very wide variation in final performance outcomes across individuals, suggesting that individual differences in response to training may be more important than the training schedule in determining performance outcomes (Scholz et al., 2009). It is therefore perhaps unsurprising that we find overlapping distributions of performance outcomes across the different practice schedules employed here. However, it is also possible that our behavioral measures are too crude to detect subtle differences, as we have not assessed juggling speed or more importantly the quality of the movement.

We restricted our primary analysis of RSNs to the motor RSN, our RSN of interest, and the default mode RSN, which we selected as a control RSN as it is largely spatially distinct from the motor RSN. However, as other recent reports have detected changes in other networks with training (Albert et al., 2009), we performed exploratory analyses (data not shown) using the same approaches described above on all networks but found no effects in any other network.

The decrease in GABA which we observed in the low intensity group after training is consistent with previous MRS studies in humans, which have shown a decrease in GABA levels in response to short-term changes in sensorimotor experience (Floyer-Lea et al., 2006; Levy et al., 2002), but the current study is the first to describe this effect after long-term learning.

The GABA findings presented here provide a putative neurophysiological basis for the functional connectivity changes demonstrated. We found a significant (though modest in strength) correlation between change in motor RSN strength and change in M1 GABA concentration due to learning. This finding offers tentative insight into the cellular mechanisms that may underlie the change in resting-state networks with motor learning and extends previous studies that have shown that the activity within the motor RSN (in the absence of learning) can be related to fluctuations in the power of beta and gamma oscillations (Brookes et al., 2011; Cabral et al., 2011), which are known to be related to GABA activity (Hall et al., 2011). In this way, our results support earlier studies (Kapogiannis et al., 2013; Stagg et al., 2014) suggesting that resting-state network strength may be driven by local GABAergic modulation of oscillatory activity within major network nodes. This study adds to these findings by suggesting that changes in RSN strength may relate to changes in GABAergic activity in response to a long-term motor training.

Interestingly, we found that GABA levels in the low intensity group had reverted to baseline by the follow-up scan, 4 weeks after the end of training. This observation is consistent with the notion that reductions in GABA are primarily found while learning is ongoing (Floyer-Lea et al., 2006). However, the return to baseline observed for our GABA measure in the low intensity group contrasts with persistent effects on functional connectivity observed in this group. These differential effects suggest that, although these two measures are related while learning is ongoing, they differ over the longer term.

These findings shed light on the processes underlying the long-term acquisition of a motor skill and, importantly, suggest that resting state fMRI may be a sensitive tool for investigating the neurophysiological changes occurring during learning and other examples of long-term plasticity such as neurorehabilitation, that can be interpreted as a type of motor learning, after stroke.

## Funding

This work was supported by the Wellcome Trust (WT090955AIA to H J-B) and FCT (SFRH/BD/43862/2008 to C S-B). CJS holds a Sir Henry Dale Fellowship jointly funded by the Wellcome Trust and the Royal Society (Grant Number 102584/Z/13/Z). The research was further supported by Marie Curie Actions (Adaptive Brain Computations network PITN-GA-2008-290011) and by the National Institute for Health Research (NIHR) Oxford Biomedical Research Centre based at Oxford University Hospitals NHS Trust and University of Oxford. The views expressed are those of the author(s) and not necessarily those of the NHS, the NIHR or the Department of Health.

**Conflict of interest statement**

No conflicts of interest are reported.
