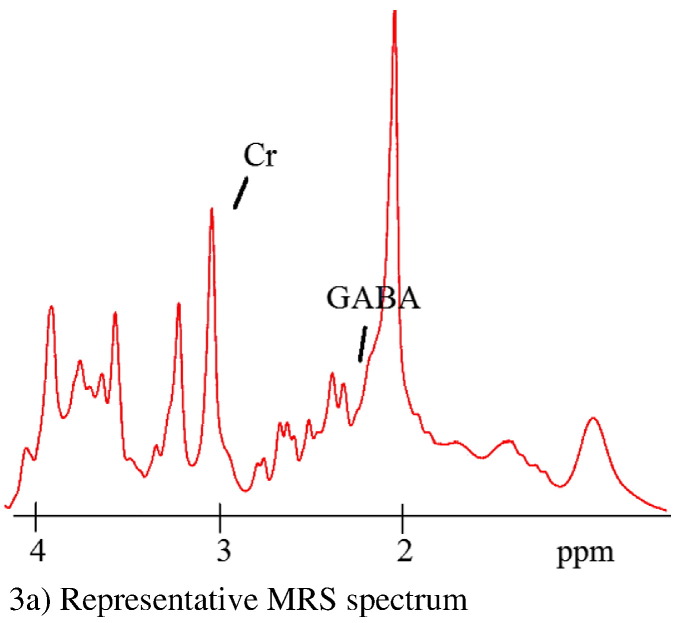# Corrigendum to “Changes in functional connectivity and GABA levels with long-term motor learning” [J. Neuroimage 106 (2015) 15-20]

**DOI:** 10.1016/j.neuroimage.2015.05.004

**Published:** 2015-08-01

**Authors:** Cassandra Sampaio-Baptista, Nicola Filippini, Charlotte J. Stagg, Jamie Near, Jan Scholz, Heidi Johansen-Berg

**Affiliations:** a Oxford Centre for Functional MRI of the Brain (FMRIB), Nuffield Department of Clinical Neurosciences, University of Oxford, John Radcliffe Hospital, Headington, Oxford OX3 9DU, UK; b Department of Psychiatry, University of Oxford, Warneford Hospital, OX3 7JX, UK; c Mouse Imaging Centre, Hospital for Sick Children, 25 Orde Street, Toronto, Ontario M5T 3H7, Canada

Dear Editor

The authors recently noticed that Fig. 3a) “Representative MRS spectrum” is incorrect as it represents a Semi-LASER MRS acquisition from 7 T, using a sequence developed by Dr. Uzay Emir and Clark Lemke. We wish to correct this figure to depict the SPECIAL MRS sequence (Mekle et al. 2009) conducted at 3 T, implemented by Dr. Jamie Near in this study. We would like to apologise for any inconvenience caused.

**Figure:**